# Protein docking prediction using predicted protein-protein interface

**DOI:** 10.1186/1471-2105-13-7

**Published:** 2012-01-10

**Authors:** Bin Li, Daisuke Kihara

**Affiliations:** 1 Department of Computer Science, Purdue University, West Lafayette, IN, 47907, USA; 2 Department of Biological Science, Purdue University, West Lafayette, IN, 47907, USA; 3 Markey Center for Structural Biology, Purdue University, West Lafayette, IN, 47907, USA

**Keywords:** protein docking prediction, protein-protein interaction, interaction site prediction

## Abstract

**Background:**

Many important cellular processes are carried out by protein complexes. To provide physical pictures of interacting proteins, many computational protein-protein prediction methods have been developed in the past. However, it is still difficult to identify the correct docking complex structure within top ranks among alternative conformations.

**Results:**

We present a novel protein docking algorithm that utilizes imperfect protein-protein binding interface prediction for guiding protein docking. Since the accuracy of protein binding site prediction varies depending on cases, the challenge is to develop a method which does not deteriorate but improves docking results by using a binding site prediction which may not be 100% accurate. The algorithm, named PI-LZerD (using Predicted Interface with Local 3D Zernike descriptor-based Docking algorithm), is based on a pair wise protein docking prediction algorithm, LZerD, which we have developed earlier. PI-LZerD starts from performing docking prediction using the provided protein-protein binding interface prediction as constraints, which is followed by the second round of docking with updated docking interface information to further improve docking conformation. Benchmark results on bound and unbound cases show that PI-LZerD consistently improves the docking prediction accuracy as compared with docking without using binding site prediction or using the binding site prediction as post-filtering.

**Conclusion:**

We have developed PI-LZerD, a pairwise docking algorithm, which uses imperfect protein-protein binding interface prediction to improve docking accuracy. PI-LZerD consistently showed better prediction accuracy over alternative methods in the series of benchmark experiments including docking using actual docking interface site predictions as well as unbound docking cases.

## Background

Many important cellular processes, such as gene expression regulation and transport, are carried out by protein complexes [1-3]. The importance and the abundance of protein interactions and complexes have been recently further highlighted by large-scale protein-protein interaction maps revealed for many organisms [4-7]. The tertiary structure of proteins is necessary for understanding the underlying molecular mechanism of protein interaction [2], however, it is often difficult to obtain complex structures by experimental methods, e.g. the X-ray crystallography or NMR. Thus, experimentally solved protein complex structures only share a small fraction among known protein complexes confirmed by biochemical experiments. Therefore, an important task in bioinformatics is to develop efficient and accurate computational methods for predicting protein-protein docking conformations.

Many protein-protein docking methods have been developed in the past employing various ideas and techniques [8-20]. Typically a docking prediction for a pair of proteins produces a few thousands of docking conformations (docking decoys), which are subject to ranking using a scoring function. Conformational search algorithms employed include the Fast Fourier Transform (FFT) [16,17,21], the Geometry Hashing [18,22], Monte Carlo algorithms [13], genetic algorithm [23,24], and Langevin dynamics [25]. For scoring a docking decoy, usually several terms are combined, which include physics-based scores [26] and those concern geometrical shape complementarity [18,27,28]. Clustering of docking decoys is also shown to be effective in selecting near native conformations [29-31]. Some of the recent docking algorithms have more elaborate procedures, for example, by considering alternative conformations of flexible protein chains [32] or post docking optimization steps [14,33]. Nevertheless, despite significant efforts of developing methods, it is still difficult to identify and rank the correct conformations in top ranks among hundreds of decoys [18,27,34] as is also evidenced by results from recent Critical Assessment of Prediction of Interactions (CAPRI), a community wide experiment on the comparative evaluation of protein-protein docking methods [10].

The accuracy of docking prediction could improve when a part, even if not all, of protein-protein interface (PPI) residues are known. PPI residues for a pair of interacting proteins can be identified by experiments including point mutation such as the alanine scanning [35-38], chemical modification of residues [39,40], NMR [41], hydrogen/deuterium exchange [42], and disulfide cross-linking [43]. If several PPI residues are known, they can be simply used for filtering, i.e. to select docking decoys which have the known PPI residues at their docking interface [44,45]. Alternatively, known PPI residues from interacting proteins can be incorporated as distant constraints [14]. However, experimental methods are time consuming. This is particularly true if identification of a whole PPI region of an interacting protein pair is attempted or if investigating many interacting proteins in a network is planned.

PPI residues can be also predicted by computational methods, which capture sequence and structural features of PPI regions [46]. There are a number of PPI site prediction methods developed. Sequence features used for PPI site prediction include amino acid residue propensity [46-52], sequence conservation [53-57], and correlated mutation [58-60]. Structure information used include hydrophobic patches, the secondary structure propensity [51], atom group propensity [61], relative accessible surface area [47], geometrical surface shape [47], the crystallographic B-factor [51], and energetic characteristics of PPI residues [62,63]. Current protein interface prediction methods choose one or combinations of these features to construct scoring functions for machine learning techniques [51,55,56,64-67]. Recent development of PPI site prediction methods has been overviewed in recent review articles [68,69]. The obvious advantage of the computational methods over experimental methods is that the former can be performed much faster than the latter. However, the problem of computational prediction methods is that they are not always accurate. For example, the Meta-PPISP method [70], one of the state-of-the-art methods, predicts PPI residues on average with a precision of 50% at the coverage of 50% for enzyme-inhibitor complexes [71]. Moreover, the prediction accuracy varies depending on target proteins and thus it is difficult to estimate the accuracy for individual cases. Therefore, computational PPI residue prediction cannot be reliably used for simple post-filtering of docking decoys. A naive use of PPI residue prediction for post-filtering may actually decrease the prediction accuracy, as we will show in Results.

Here, we present a novel protein docking algorithm, PI-LZerD (using Predicted Interface with Local 3D Zernike descriptor-based Docking algorithm), which utilizes imperfect PPI residue prediction for guiding protein-protein docking. PI-LZerD performs iterative improvement of docking results starting from an initial run of docking that uses potentially inaccurate PPI prediction as restraints. The base of the docking algorithm used is the LZerD (Local 3D Zernike descriptor-based Docking algorithm), which we have developed previously [18]. The idea of using additional predicted information for aiding protein docking has been explored by a few previous works. In their works, PPI information is used for post-filtering docking decoys [16,71-73] or incorporated as an additional scoring term [14,45,74,75]. Compared to these related works, the current work is significantly different in the design and some important aspects: First, we have developed a novel algorithm which is specifically designed to utilize imperfect PPI prediction. Thus, we don't use PPI information simply for post filtering. Second, we perform thorough investigation on how the accuracy of PPI prediction affects to the docking prediction accuracy. PI-LZerD is shown to be able to consistently improve docking predictions when actual PPI predictions are used for unbound docking cases. The datasets used and the developed PI-LZerD program are made freely available for academic community.

## Methods

### Pairwise protein docking algorithm, LZerD: the original algorithm

We start with brief explanation of the original LZerD pairwise protein-protein docking algorithm [18]. As will be explained in the next section, PI-LZerD performs an iterative use of a modified version of LZerD. LZerD takes two protein tertiary structures (Protein Data Bank, PDB [76], files) as input (termed a ligand and a receptor protein) and outputs over 30000~50000 of docking decoys ranked by a scoring function. The geometric hashing algorithm [77] is used for docking conformational search.

**Figure 1:**
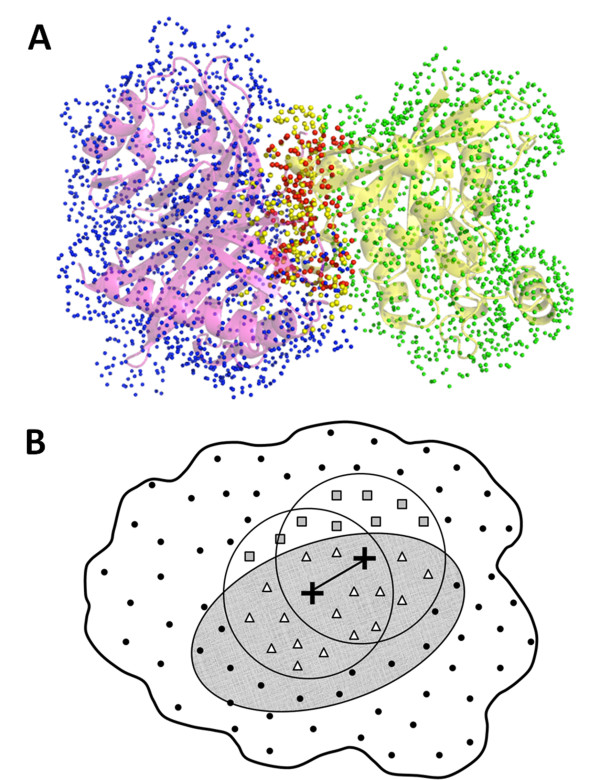
**Surface points used in geometric hashing**. **A**, Example of surface points. Surface points of 1A2K chain B (left, purple, 248 residues) are colored in blue for non-PPI surface (1652 points) and yellow for the PPI region (25 residues, 248 points). Critical points of 1A2K chain C (right, yellow, 196 residues) are colored in green for non-PPI surface (1332 points) and red for the PPI region (16 residues, 165 points). **B**, Two schemes for restricting docking interface using predicted PPI regions in geometric hashing. Two base points are selected from points that locate within the predicted PPI regions (gray ellipsoid). Given the two base points (crosses), points in their neighborhood (within 15Å; showed in two circles) are transformed based on the coordinate system defined by the base points, which are then stored in a hash table. Fitness of local regions around the base points of the ligand and the receptor proteins are evaluated by the number of equivalent matching points (the voting stage). In a permissive search, points from outside of the PPI region (squares) as well as points within the PPI region (triangles) are considered. For a restrictive search, only the points in the PPI region (triangles) are considered.

Given a protein tertiary structure, protein surface is constructed and then points are distributed evenly on the surface. Typically, about 1500~2000 points are distributed for a 200-250 amino acid long protein (Figure 1A). The geometric hashing procedure pre-computes and records all possible orientations of the ligand protein (the hashing stage). This is done by defining a coordinate system for each pair of surface points (called base points), based on which the coordinates of the other neighboring surface points (within 15Å to the base points) are computed. Note that a three dimensional coordinate system can be uniquely defined by two points by using the average of normal vectors of the two points as additional parameter. Once all the poses of the ligand protein are recorded in a hash table, each possible pose of the receptor protein is computed in the same way, which are then compared with the poses of the ligand protein stored in the hash table (the matching stage). If a sufficient number of points from the two proteins match (voting stage), the conformation is further evaluated by a physics-based score.

The scoring function is a weighted sum of the following terms: van der Waals, where, repulsive and attractive parts of the term are considered separately [13]; an electrostatics term, which considers repulsive/attractive and short-range/long-range contributions separately [78]; a hydrogen and disulfide bond term [79]; two solvation terms [80,81]; and a knowledge-based atom contact term [82]. Weighting factors for the linear combination of the terms were trained on two datasets, the protein-protein docking benchmark 2.0 [83], which contains 84 pairwise unbound-unbound and bound-unbound docking structures, and also on 851 protein-protein dimeric complexes compiled by Huang and Zou [84]. The combination of weight values were determined by using logistic regression with the interface root mean square deviation (iRMSD) between predicted decoys and the native structure as the target function to be optimized.

### Modified LZerD to incorporate PPI prediction

We modified the LZerD algorithm so that additional information of a PPI region can be used to restrict the docking search space. Figure 1B illustrates the two methods of restricting conformational search space in geometric hashing. Given a set of (predicted) PPI residues in a ligand or a receptor protein, each surface point is classified into either PPI (points within the gray ellipsoid in Figure 1B) or non-PPI depending on whether the closest atom for the point belong to a PPI residue or not. In the geometry hashing, two base points (two crosses) are selected to define a reference coordinate system, based on which the other local points are transformed. Base points are selected only from the PPI surface points for both ligand and receptor proteins. Then, in the voting stage, matching points between the ligand and receptor are counted either only from the PPI surface points (i.e. matches are only considered within the predicted PPI regions; triangles in the region in gray in Figure 1B) or from all the surface points (triangles and squares) including non-PPI points. Obviously, the former seeks for a geometrical complementarity of the two proteins only at the predicted PPI regions while the latter explores a wider surface area to identify well fitting docking conformation in the vicinity of the predicted PPI regions. PI-LZerD uses these two search areas in different stages of docking conformation search. The more permissive search area is considered for the initial LZerD run and the more restricted searches are performed for the subsequent iterations.

### PI-LZerD algorithm

**Figure 2:**
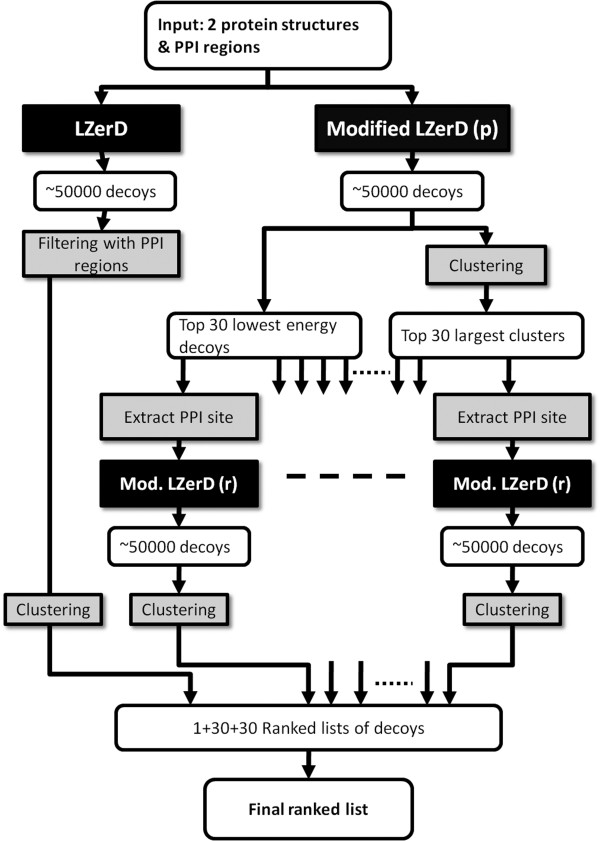
**Overview of the PI-LZerD algorithm**. See the text for the explanation. "LZerD" is the original version of the LZerD docking program. Modified LZerD (p) and (r) stands for the permissive search space and the restrictive search space employed in the geometric hashing stage, respectively.

The PI-LZerD algorithm performs pairwise protein-protein docking prediction using additional information of PPI residues as constraints. The algorithm is illustrated in Figure 2. First, given the tertiary structure and (predicted) PPI regions of the two proteins to be docked, the modified LZerD is run to yield typically 30000~50000 docking decoys (the right branch of the diagram). For this initial run of the modified LZerD, all neighboring surface points to the base points are considered in the voting stage (the permissive search as discussed in the previous section). The docking decoys are ranked by the physics-based score and top 1000 best scoring decoys are selected.

The 1000 decoys are subject to clustering by considering the similarity of docking interface regions. For a given pair of docking decoys, common atoms between the two PPI regions from the two decoys are selected. Then, the RMSD is computed for the common atoms only when the common atoms share more than 60% of all interface atoms of both PPI sites (if the common atoms do not exceed 60% then the two proteins are not clustered together). We call it the common interface RMSD (ciRMSD) of two docking decoys. The ciRMSD is more suitable for the PI-LZerD algorithm as compared to the conventional coordinate RMSD [85] or the ligand RMSD [86], since it focuses on capturing the similarity of docking interface regions.

Once the ciRMSD is computed for all the pairs of decoys, 60 decoys are selected by considering the physics-based score and the cluster size of the decoys. First, the decoy with the lowest score (the lower, the better) is selected and close decoys with a ciRMSD ≤ 4.0Å are discarded from the pool. This process is repeated until 30 decoys are identified. Next, additional 30 decoys are selected based on the cluster size. For each of the decoys, the number of the other decoys within 4.0Å ciRMSD is computed. Then, the largest cluster (i.e. the center decoy with the largest number of close decoys) is selected. If several clusters with the same size are found, the one which has the center decoy with the lowest physics-based score is selected. All the decoys in the cluster are removed, and the process is repeated until 30 representative decoys are selected. Consequently, 30 decoys are selected based on the lowest energy and 30 more decoys are selected based on the cluster size. It was shown that combining the energy value and the cluster size can find more hits than using a single metric alone (Additional file 1, Figure S1).

The selected 60 decoys are passed to the subsequent process. For each of the 60 docking decoys, PPI residues are extracted. PPI residues are defined as those which have a heavy atom closer than 5.0 Å to any atom to the docking partner. The decoys do not necessarily have the identical PPI region as the initially provided PPI information because the modified LZerD has explored the vicinity of the input PPI in the docking conformation search. Using the identified PPI residues as the updated constraint, the modified LZerD is run for the second time. In this round, only the PPI surface points are considered at the voting stage in the geometric hashing (the restrictive search). From the resulting docking decoys, the top 1000 lowest energy docking decoys are clustered based on ciRMSD, whose cluster centers are sorted by the physics-based score. Since the modified LZerD is run for each of the 60 decoys, in total of 60 LZerD runs are performed.

In addition to the 60 runs of the modified LZerD, we run the original LZerD without using predicted PPI information followed by post-filtering by using the predicted PPI residues (naive-filtering method) (the left branch of Figure 2). In the naive-filtering method, docking decoys are sorted not by the physics-based score but by the agreement of the docking interface residues to the predicted PPI residues. Therefore, the overall procedure produces 61 runs of docking predictions, i.e. 61 ranked lists of docking decoys. To make the final ranking of docking decoys, first, the top ranked docking decoys from each of the 61 lists are ranked by the physics-based score, and then the decoys in the same subsequent ranks from the 61 lists are ranked in the same way. Thus, the decoys from all the lists are first sorted by their ranks in each list then sorted by the physics-based score. If the identical decoys appear, one which is ranked lower in the entire final list is removed (it is not common but possible that identical docking decoys appear in different LZerD runs).

### Dataset of protein complexes and PPI information

The first dataset we use for benchmarking PI-LZerD is the protein-protein docking benchmark version 3.0 [87] with 124 bound cases. The average length of the proteins is 256 and the number of docking interface residues of the proteins range from 10 to 70 with an average of 25.

To investigate how the accuracy of PPI prediction affects to the docking prediction, we first use "simulated" PPI predictions as input. The actual PPI region of a ligand and a receptor proteins are shifted by 5, 10, 12, and 15 residues to two opposite directions on the protein surface along the major axis of the PPI region. To shift a PPI region on the surface, *n *PPI residues (*n *= 5, 10, 12, 15) at one end of the PPI site along the axis are removed and the same number of residues are added on the opposite side of the PPI site. Thus, the shifting of PPI regions are done geometrically rather than along the protein sequence (Additional file 1, Figure S2). By combining two shifted PPI regions from a ligand and a receptor protein, four test cases are made for each protein complex (because the PPI region on each protein is shifted in two opposite directions). The protein complexes are removed from the dataset if one of proteins has a smaller PPI region than the number of shifted residues. The total number of tested protein complexes with 5, 10, 12, and 15 PPI residues shift are 124 (124 × 4 = 496 test cases), 122 (488 cases), 118 (472 cases), and 104 (416 cases), respectively. Since four different combinations of shifted PPI regions of a ligand and a receptor are tested, the number of tested cases is four times of the number of protein complexes, which is shown in the parentheses.

**Figure 3:**
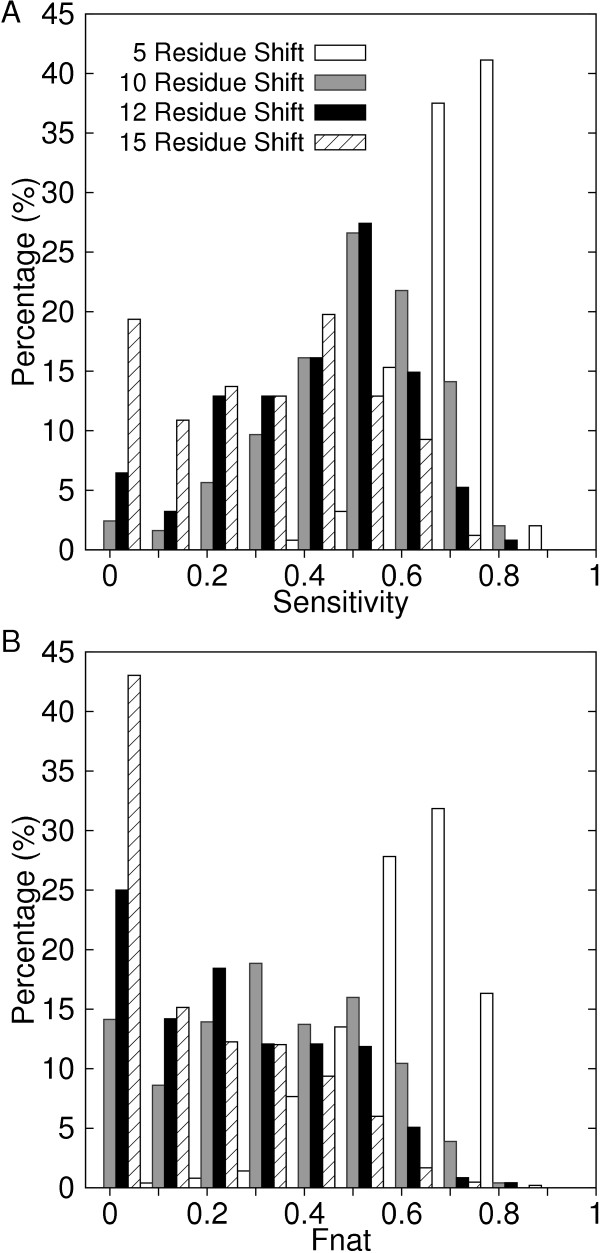
**Accuracy of the shifted PPI regions**. Distributions of **A**, the sensitivity (= specificity); **B**, the fnat value; of the shifted PPI regions by 5, 10, 12, and 15 residues.

Figure 3A shows the distribution of the sensitivity of shifted PPI regions (i.e. the fraction of the correctly predicted PPI residues among actual PPI residues). The sensitivity depends on the size of the proteins PPI regions for the same number of residues shifted. The average sensitivity for the 5, 10, 12, and 15 residue shifted PPI sites are 0.767, 0.535, 0.447, and 0.324, respectively. The specificity value (the fraction of correctly predicted PPI residues over the total number of predicted PPI residues) is the same as the sensitivity because the size of a shifted PPI region is the same as the actual one. In Figure 3B, the fraction of correctly interacting PPI residue pairs in the protein complexes with shifted PPI regions is shown (the fraction of the native contacts, fnat [88]). The fnat value depends on the shifting directions even for the same pair of shifted PPI sites. The average fnat value for proteins pairs with 5 residue shifted PPI is 0.673. The fnat value distributes more broadly for more shifted PPI sites. The average PPI sites for 10, 12, 15 shifted residues are 0.364, 0.275, and 0.191.

We also test PI-LZerD using actual PPI predictions with a state-of-the-art PPI prediction method, meta-PPISP [70]. Meta-PPISP is a meta server which combines predictions by three methods, Promate [51], PINUP [49], and cons-PPISP [54]. The benchmark dataset is selected from the iPFAM database [89], a subset of PFAM database [90], which provides multiple sequence alignments (MSA) of interacting proteins. We used iPFAM because meta-PPISP needs a MSA as an input. The iPFAM entries were pruned using the following criterion: (1) PFAM families with 20 to 100 seed sequences were selected. (2) PFAM families consisting local domain sequences were replaced with their corresponding full-length sequences from UniProt [91]. A representative PDB structure was then selected from each PFAM family given by the association in iPFAM. (3) Protein structures that do not have any observable interacting partners in their PDB files were removed. (4) Proteins with their PDB entries that have non-standard amino acids and obsolete PDB files were filtered out. (4) PDB structures with antibody-antigen and protein-DNA/RNA interactions were removed. (5) Protein complexes with more than two chains are removed. (6) Complexes were eliminated if they are classified as monomers bound by crystal contacts in the PQS definition [92]. (7) Proteins with the size between 75 to 300 amino acids were selected. (8) In the final dataset, PFAM families with redundant representative structures with ≥35% sequence identity were filtered out. Given that MSAs in PFAM may not have the PDB structure as a part of the alignment, we employed MUSCLE (ver. 3.6) [93] with default parameters to compute MSAs from PFAM unaligned sequences and one sequence from the selected PDB structure. The final dataset includes 127 protein complexes. Using prediction output of the meta-PPISP server, residues which have a meta-PPISP score of 0.1 or higher are identified as PPI residues.

### Availability and requirements

The executable program of PI-LZerD for Linux is freely available to academic institutions at our website, http://kiharalab.org/PI-LZerD. The datasets used in this study are also available at the same webpage. The program requires a computer with at least 1.5 GB RAM operated by Linux OS. The average times combining both docking and scoring range are about 1-2 hours for small proteins (about 400 points on the receptor and ligand) and it may take longer for larger proteins. This timing is reported on a computer with a dual-core 2.1 GHz processor with 8 GB RAM. In addition, the pairwise docking program, LZerD, which is the base of PI-LZerD, is also made available at http://kiharalab.org/proteindocking.

## Results and Discussion

### Naive post-filtering method

An obvious approach to use predicted PPI information for protein docking prediction is to select docking decoys with a PPI site that agrees well to the provided PPI information. This approach, termed as the naive post-filtering method, was tested on datasets with the five different accuracy levels of PPI prediction. In addition to the set of accurate PPI information, we used PPI sites shifted by 5, 10, 12, and 15 residues. For each protein complex with PPI information, we run original LZerD to produce top 1000 scoring docking decoys. Then, for each docking decoy, the fraction of the overlap of residues in the provided PPI information the PPI region of the docking decoy is computed for both ligand and for the receptor proteins, and the average of the two are used for sorting decoys.

**Figure 4:**
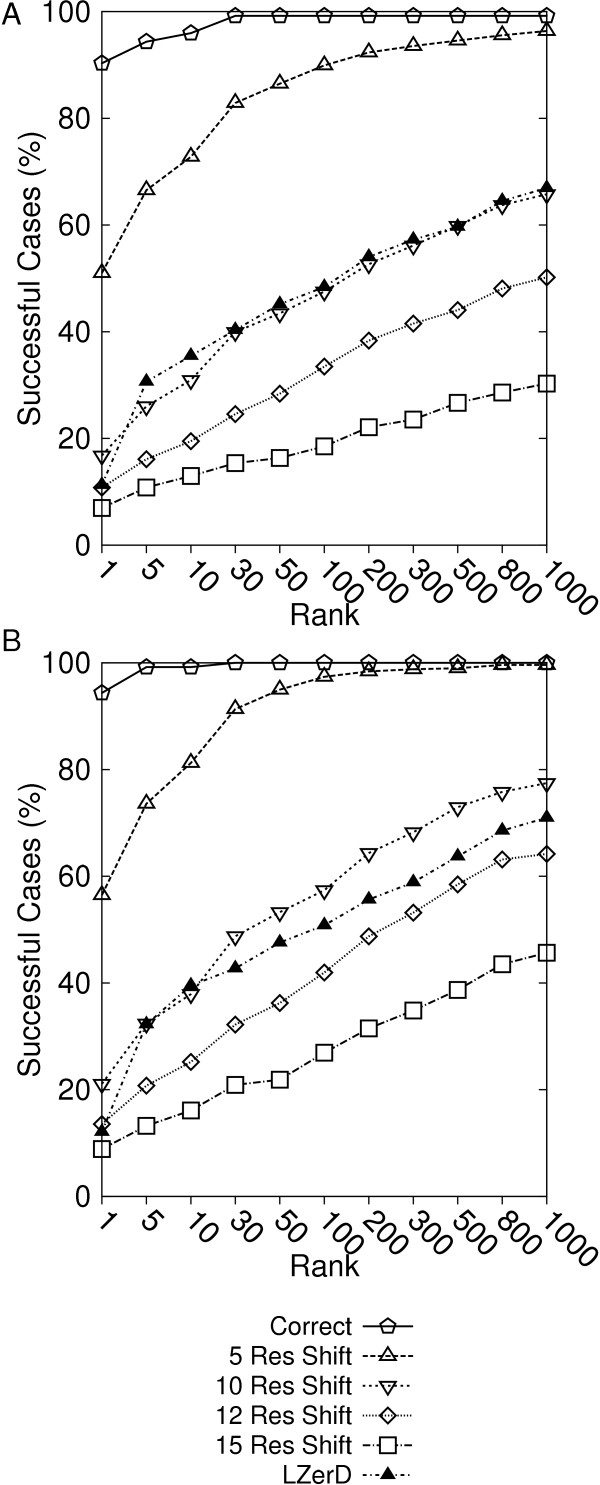
**The prediction accuracy of the naive post-filtering method**. The percentage of the cases among the tested complexes is shown where the naive post-filtering method obtained a near native structure of below **A**, 2.5 Å iRMSD; **B**, 4.0 Å iRMSD; within specified ranks at the x-axis. PPI site information of five different accuracy levels are used: correct PPI (pentagons); 5 residue shifted (downward triangles); 10 residue-shifted (upward triangles), 12 residue-shifted (diamonds); and 15 residue-shifted PPI regions (squares). For comparison, results of the base LZerD which do not use PPI information are also shown (filled triangles).

In Figure 4, the fraction of the protein complexes where correct prediction (interface RMSD ≤ 2.5Å in Figure 4A; 4.0Å in Figure 4B) exist within specified ranks cutoff (x-axis) are shown. The prediction accuracy of original LZerD without using the PPI information is also shown for comparison. The naive post-filtering achieved near perfect prediction accuracy when the perfectly accurate PPI information was provided. When the PPI information was shifted by five residues, the prediction accuracy at top 1 rank dropped significantly, from 90% to 51% when iRMSD of 2.5 Å is used as the threshold (Figure 4A). Interestingly, using further deteriorated PPI information of ten residues shift made prediction results indistinguishable from running LZerD without PPI information. Using more inaccurate PPI prediction of twelve or fifteen residue shifts was shown to be even harmful, producing worse predictions than LZerD without PPI information. The consistent trend was observed in Figures 4A and 4B. Overall, the results show that the naive post-filtering is sensitive to the accuracy of PPI residue information used for the filtering. Thus, it is not reliable to apply PPI prediction simply for post-processing when the accuracy of the prediction is not well known.

### PI-LZerD with simulated PPI predictions

Next we examine performance of PI-LZerD on the dataset of simulated PPI predictions. This experiment is for understanding the effect of various levels of inaccuracy in PPI predictions to the docking results. In the later sections we discuss the results using actual PPI predictions on bound and unbound docking cases. The full implementation of PI-LZerD (Figure 2, PI-LzerD-2) was compared with four other variations of LZerD, namely, the original LZerD without PPI information (the base LZerD), the original LZerD followed by post-clustering without using PPI information, LZerD with naive post-filtering with the PPI information, and PI-LZerD using PPI information with only one iteration of the modified LzerD (PI-LZerD-1). PI-LZerD-1 clusters output of docking decoys using the ciRMSD.

**Figure 5:**
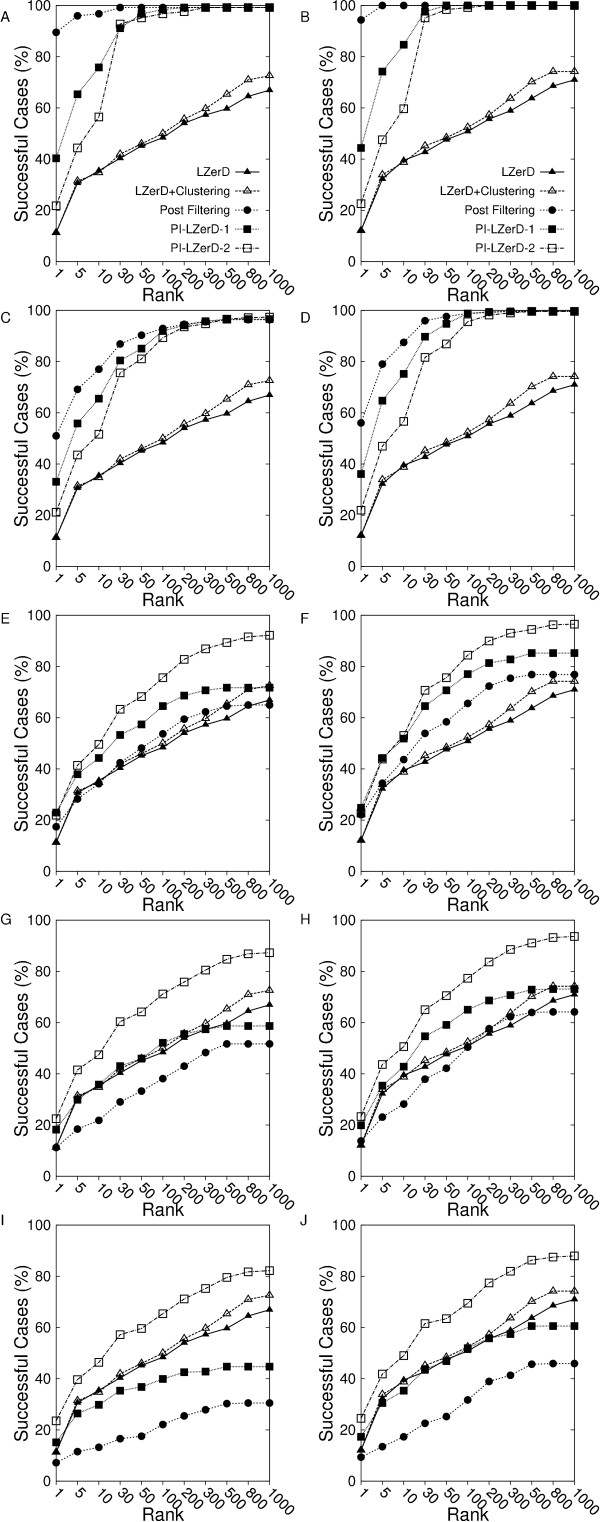
**Docking prediction with simulated protein interface predictions**. Four other methods are listed to compare with PI-LZerD-2: the base LZerD (LZerD), LZerD with clustering using ciRMSD (LZerD+Clustering), LZerD with one interaction of modified LZerD (PI-LZerD-1), and Simple residue filtering method (post-filtering). The x-axis indicates the ranks in logarithmic scale and the y-axis shows the percentage of cases where correct predictions are ranked equal or better than the corresponding ranks. Left panels, **A, C, E, G, I**, use the 2.5 Å as threshold for correct predictions, while right panels, **B, D, F, H, J**, use 4.0 Å as the cutoff for near hit predictions. **A **&**B **use the correct protein interface information; **C/D **use the simulated predictions with 5 residue shifts, **E/F**, **G/H**, and **I/J **use the simulated predictions with 10, 12, and 15 residue shifts, respectively.

Figure 5 shows prediction results of the five methods using 0, 5, 10, 12, and 15 residue shifted PPI information. The y-axis shows the fraction of successful cases where a correct prediction exists within specified ranks cutoff on the x-axis. When the provided PPI residues are 100% accurate, the naive post-filtering can naturally select correct predictions among the pool of docking decoys (Figures 5A &5B). PI-LZerD with one or two iterations performs better than the base LZerD. Since PI-LZerD does not restrict the conformation search space to the provided PPI site but also explores its neighborhood, PI-LZerD obtained a hit for a less number of complexes within top 30 ranks than the naive post-filtering method. However, when top 100 ranks are considered, both naive post-filtering and PI-LZerD-2 and PI-LZerD-1 made successful prediction for almost all the tested cases. The clustering step made a slight improvement of accuracy when applied to decoys generated by the base LZerD.

As the accuracy of the PPI information starts to deteriorate, the docking prediction accuracy by the naive post-filtering quickly drops relative to the others. When 5 residue shifted PPI information was used, the post-filtering method still showed the highest number of successful cases up to the 100 ranks (Figures 5C &5D). When PPI regions were further shifted by 10 residues, PI-LZerD clearly outperformed the post-filtering method. The performance of the post-filtering method went down as low as the base LZerD which did not use the PPI information. It is also noticed that the PI-LZerD-2 performed better than PI-LZerD-1.

Figures 5G &5H show that when the 12 residue shifted PPI regions were used, the naive filtering method performed even worse than the base LZerD. In contrast, remarkably, PI-LZerD-2 managed to successfully use the inaccurate PPI information, showing a higher accuracy than the base LZerD. The accuracy of PI-LZerD-1 is now comparable to the base LZerD when 2.5 Å iRMSD threshold was used (Figure 5G) but better for 4.0 Å iRMSD threshold (Figure 5H). Finally, with 15 residue shifted PPI regions (Figures 5I &5J) PI-LZerD-2 still remained superior to the base LZerD while the accuracy by the naive post-filtering went further down. It is worth mentioning that the prediction accuracy by PI-LZerD-2 stays almost the same with 5, 10, 12, and 15 shifted PPI regions. Importantly, the stability of the prediction by PI-LZerD was observed only for PI-LZerD-2 but not PI-LZerD-1. This indicates that the two iterations of modified LZerD run are necessary to effectively explore the vicinity of specified PPI region to find the lowest energy conformation.

In Additional file 1, Figures S3 and S4, we analyzed the same results by classifying the shifted PPI sites by their accuracy. In Additional file 1, Figure S3, the protein complexes are classified by the average sensitivity of the shifted PPI sites of the receptor and the ligand proteins, while they are classified based on the fnat of shifted PPI sites of the receptor and the ligand proteins in Additional file 1, Figure S4. Essentially the same trend was observed in Additional file 1, Figures S3 & S4 as Figure 5. Using the naive post-filtering, near perfect prediction accuracy can be achieved only when the correct PPI information is provided. However, its results quickly deteriorate as the accuracy PPI site information drops. In contrast, PI-LZerD can take advantage of PPI information even when it is not very accurate. For the range of the PPI site information accuracy tested, PI-LZerD always showed better performance than the base LZerD without using PPI information. It is very important that employing additional information (in this case PPI site prediction) do not deteriorate prediction results even if the quality of information is not high, which is successfully achieved by PI-LZerD.

### Docking Prediction using actual PPI site prediction

**Figure 6:**
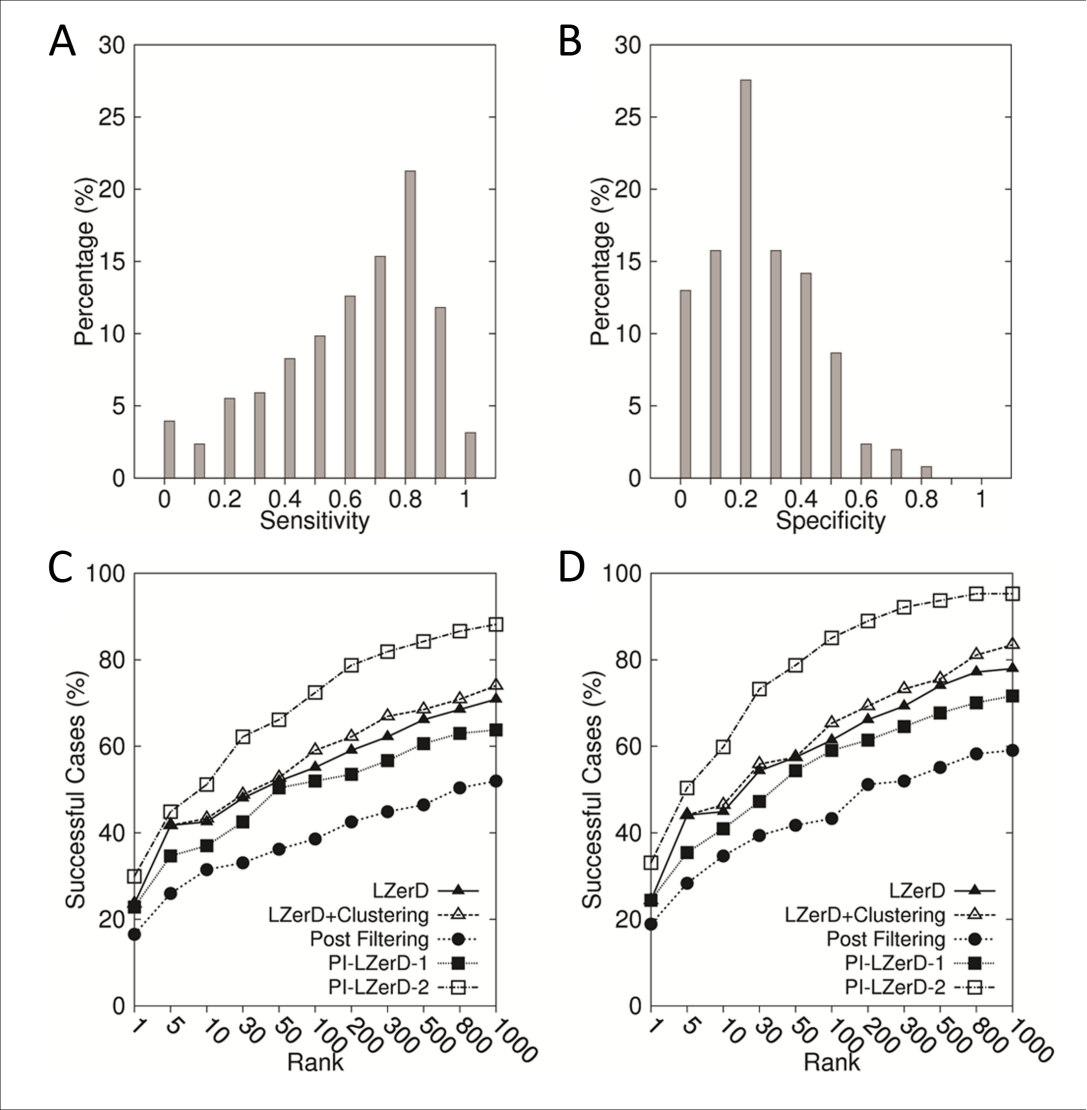
**Docking prediction results using meta-PPISP binding site prediction**. The meta-PPISP server predicted PPI regions of 127 complexes selected from the iPFAM database. Distribution of **A**, the sensitivity and **B**, the specificity of the meta-PPISP prediction. The docking prediction results using **C**, 2.5 Å iRMSD cutoff; **D**, 4.0 Å iRMSD to define correct predictions.

Next, we use actual PPI site prediction for PI-LZerD. 127 protein complexes taken from the iPFAM database were used in this experiment. PPI site predictions were computed by Meta-PPISP [70] using MSAs taken from the iPFAM database. The average sensitivity and the specificity of the prediction by meta-PPISP were 0.648 and 0.297, respectively (Figures 6A &6B), when the cutoff score of 0.1 was used. The average sensitivity value is better while the specificity value is worse than the simulated PPI site predictions we used in the previous section, which were 0.535 for both sensitivity and the specificity.

On this dataset, PI-LZerD-2 performed consistently the best at every rank cutoff (x-axis) with both 2.5 Å and 4.0 Å (Figures 6C &6D) iRMSD thresholds. Within top 10 predictions, PI-LZerD-2 made at least one hit for 51.2% of the cases, while the base LZerD and the naive post-filtering obtained hits for 42.5%, 31.5% of the cases with the 2.5 Å iRMSD cutoff (Figure 6C). Within the rank of 100, the successful cases for the methods increased to 72.4, 55.1, and 38.6%, respectively. Thus, PI-LzerD-2 improved the success rate over the base LZerD by 8.7 and 17.3% points within the rank of 10 and 100. When 4.0Å is used for iRMSD cutoff (Figure 6D), PI-LZerD-2 obtained at least one hit for 33.1/59.8/85.0/95.3% within top 1/10/100/1000 predictions, respectively. The naive post-filtering performed consistently worse than the base LZerD. An important conclusion from these results is that blind PPI site predictions cannot be used for improving docking prediction with the post-filtering procedure. On average it will only deteriorate prediction accuracy.

### Unbound protein docking using actual PPI site prediction

**Figure 7:**
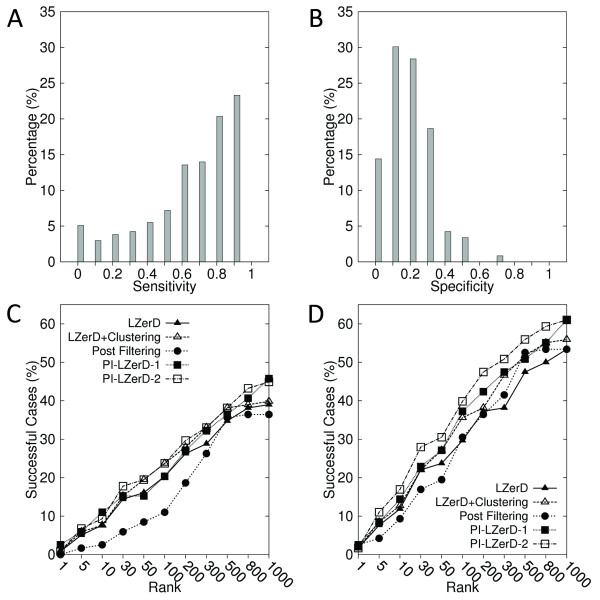
**Docking results on the unbound dataset using meta-PPISP prediction**. 118 unbound-unbound protein complexes were taken from the docking benchmark dataset version 3.0. The distribution of **A**, the sensitivity; and **B**, the specificity. **C **and **D **use 2.5 Å and 4.0 Å, respectively, for the iRMSD cutoff value to define correct prediction.

We have further benchmarked PI-LZerD on unbound docking cases. Out of 128 unbound docking cases in the protein-protein docking benchmark dataset 3.0 [87], 118 cases were selected that are not longer than 800 residues and have an MSA in the iPFAM database. The PPI sites were predicted by the meta-PPISP server. The iRMSD between bound and unbound complexes of this dataset ranges from 0.17 Å to 8.38 Å with an average of 1.34 Å. Figures 7A &7B provide the distribution of the sensitivity and the specificity of the PPI site predictions by meta-PPISP. The average value was 0.684 and 0.231 for the sensitivity and the specificity, respectively.

We observe again the same trend as we observed in the previous experiments: PI-LZerD-2 showed consistently better success rate than the base LZerD at each rank cutoff (Figures 7C &7D). At the rank cutoff of 10, 100, 1000, PI-LZerD-2 made successful predictions within 2.5 Å iRMSD (Figure 7C) for 9.32%, 23.73%, and 44.92% of the cases, while the success rate of the base LZerD was 7.63%, 20.34%, and 38.98%. With 4.0 Å iRMSD cutoff, (Figure 7D), the success rate of PI-LZerD-2/the base LZerD was 16.95/11.86, 39.83/29.66, and 61.02/53.39 at 10, 100, 1000 ranks. The naive post-filtering performed again worse than the base LZerD at most of the rank cutoff values.

**Figure 8:**
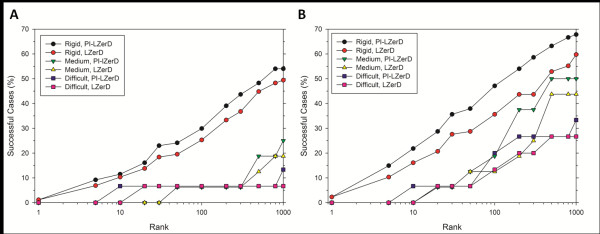
**Docking results on the unbound dataset using meta-PPISP prediction are classified by the docking difficulty**. The same results shown in Figure 7 are classified into three docking difficulty classes, rigid-body (87 cases), medium (16 cases), and, difficult (15 cases). **A**, **B**, results using 2.5 Å and 4.0 Å, respectively, for the iRMSD cutoff value to define correct prediction. Black/red circles, the rigid-body category results by PI-LZerD/LZerD; green/yellow triangles, the medium category by PI-LZerD/LZerD; Black/red squares, the difficult category by PI-LZerD/LZerD.

The same prediction results are categorized according to the three difficulty levels for protein docking assigned by the docking benchmark dataset. The 118 unbound cases contain 87 rigid-body docking cases, 16 medium cases, and 15 difficult cases. In Figure 8, the success rates for the three difficulty levels are separately shown. For all the three levels, PI-LZerD consistently showed a higher or equal success rate as compared LZerD. The improvement by PI-LZerD over LZerD is more evident when 4.0 Å iRMSD cutoff is used (Figure 8B).

Using this test set, we have also examined the effect of using a different number of decoys in the second round of LZerD run in PI-LZerD. As shown in the illustration of the PI-LZerD algorithm (Figure 2), we use top 30 lowest energy decoys and another 30 decoys with the largest clusters, thus 60 decoys, as the sources of updated PPI sites. We compared prediction results using 50 (i.e. 25 lowest energy decoys and 25 largest cluster decoys), 80, and 100 decoys in Additional file 1, Figure S5. The results show that using 60 docking decoys performs overall best among tested when the cutoff of 2.5 Å is used. When the cutoff of 4.0 Å is used to define near native decoys, all of them showed similar performance.

### Examples of docking prediction by PI-LZerD

**Figure 9:**
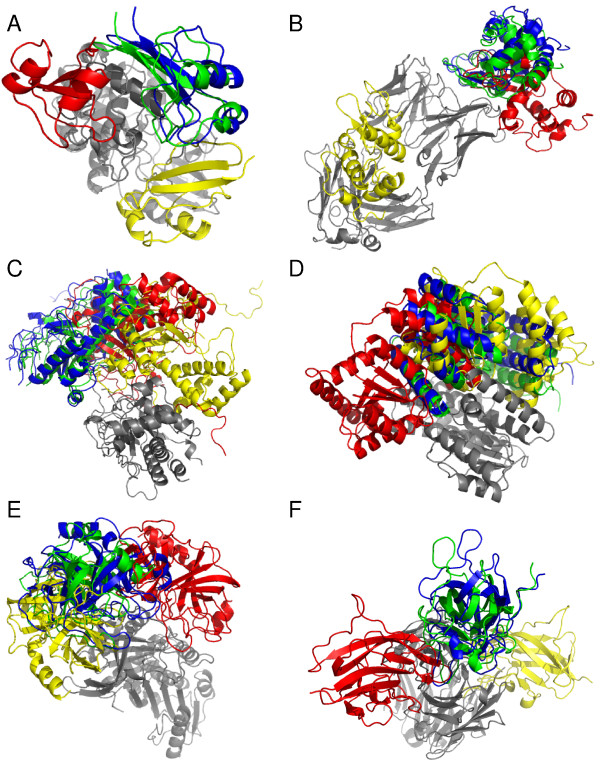
**Examples of docking prediction**. The best prediction within 50 ranks by PI-LZerD-2, the base LZerD, and naive post-filtering is shown in green, yellow, and red, respectively. The correct docking pose is shown in blue. Receptor proteins are shown in gray. First, predictions using 10 residue-shifted PPI site information are shown for **A**, 1BUH; **B**, 1MLC. For 1BUH, the iRMSD (Å) of prediction by PI-LZerD-2/post-filtering/base LZerD was 1.03/9.09/9.91. The rank of the decoys was 8/24/17. The sensitivity (= specificity, since the size of the shifted PPIs are same as the correct PPI) of the shifted PPI region was 0.33 for 1BUHA and 0.44 for 1BUHB. For 1MLC, iRMSD (Å) and the rank of the best prediction within 50 ranks by PI-LZerD-2/post-filtering/base LZerD were 0.89 Å (34)/8.37 Å (9)/14.35 Å (22). The ranks are shown in the parentheses. The sensitivity (= specificity) of the shifted PPI region was 0.55 for chain A and B, and 0.44 for chain E. **C **and **D **are predictions using actual PPI site predictions by meta-PPPISP for proteins from the iPFAM dataset. **C**, 1ADU. The iRMSD (Å) and the rank of the predictions by PI-LZerD-2/post-filtering/base LZerD were 1.04 Å (48)/14.90 Å (37)/10.85 Å (43). The sensitivity/specificity of PPI site predictions were 0.77/0.47 for 1ADUA, and 0.00/0.00 for 1ADUB. **D**, 1BMT. The iRMSD (Å) and the rank of the predictions by PI-LZerD-2/post-filtering/base LZerD were 2.31 Å (36)/14.44 Å (31)/13.02 Å (44). The sensitivity/specificity of PPI site predictions were 0.11/0.06 for 1BMTA, and 0.22/0.11 for 1BMTB. The last two examples are unbound docking cases with actual PPI predictions by meta-PPISP. **E**, 1OPH. The iRMSD (Å) and the rank of the predictions by PI-LZerD-2/post-filtering/base LZerD were 3.76 Å (42)/5.71 Å (39)/10.28 Å (16). The sensitivity/specificity of PPI site predictions were 1.00/0.32 for 1OPHA, and 0.70/0.18 for 1OPHB. **F**, 1IQD. The PI-LZerD-2/post-filtering/base LZerD: 2.91 Å (23)/6.97 Å (16)/12.80 Å (32). The sensitivity/specificity of PPI site predictions were 0.53/0.16 for 1IQDA, and 0.89/0.20 for 1IQDB.

Here we show examples of docking predictions that illustrate difference of PI-LZerD as compared to the base LZerD and the naive post-filtering. The first two examples (Figures 9A &9B) are from prediction using simulated PPI predictions. For all the cases actual PPI regions were shifted by 10 residues. The best iRMSD structures within top 50 decoys are shown.

First example is human cdk2 kinase complex with cell cycle-regulatory protein ckshs1 (PDB ID: 1BUH). The best predictions within top 50 using PI-LZerD/naive post-filtering/LzerD were 1.03 Å (8)/9.09 Å (24)/9.91 Å (17) iRMSD, respectively. In the parentheses the rank of the decoys are shown. The second example (Figure 9B) is monoclonal antibody fab d44.1 complexed with lysozyme (1MLC). The best prediction using PI-LZerD-2/naive post-filtering/LZerD were 0.89 Å (34)/8.37 Å (9)/14.35 Å (22) iRMSD, respectively. The predicted ligand protein position by the naive post-filtering method (shown in red) indicates where the shifted PPI site information pointed. Thus, PI-LZerD managed to find the near native docking pose (green) from the originally provided wrong PPI site information. The near native pose (iRMSD ≤ 4.0 Å) was not found among the top 50 lowest energy score decoys.

The next two examples are taken from the iPFAM dataset where actual PPI predictions by meta-PPISP were used (Figure 6). Figure 9C is a complex of adenovirus single-stranded DNA-binding proteins (1ADU). The PPI site prediction by meta-PPISP is fine for one protein (sensitivity: 0.77) but totally missed the correct PPI site for another protein (sensitivity and specificity of 0.0). PI-LZerD-2 managed to identify a 1.04 Å iRMSD conformation (blue) while the naive post-filtering method made significantly wrong prediction (red). The LZerD energy function failed to identify the near native conformation within top 50 ranks (yellow). Figure 9D is a complex of methionine synthase (1BMT). The best PI-LZerD-2 prediction is at 2.31 Å iRMSD, while the post-filtering method and the base LZerD predictions are at iRMSD of 14.4 Å and 13.0 Å iRMSD, respectively. The PPI prediction for the both chains are much worse than average.

The last two examples are from unbound docking experiments using meta-PPISP predictions. The first example is the predictions for α-1-antitrypsin precursor and trypsinogen complex (1OPH). The best iRMSD predictions by PI-LZerD, the post-filtering, and base LZerD were 3.76 Å, 5.71 Å, and 10.28 Å, respectively. The last one, the complex of human factor VIII and human monoclonal BO2C11 Fab (1IQD), again PI-LZerD-2 identified a near-native pose (an iRMSD of 2.91 Å) (Figure 9E). The base LZerD found lower energy decoys at very different position, an iMRSD of 10.28 Å.

### Comparison with an existing method

**Figure 10:**
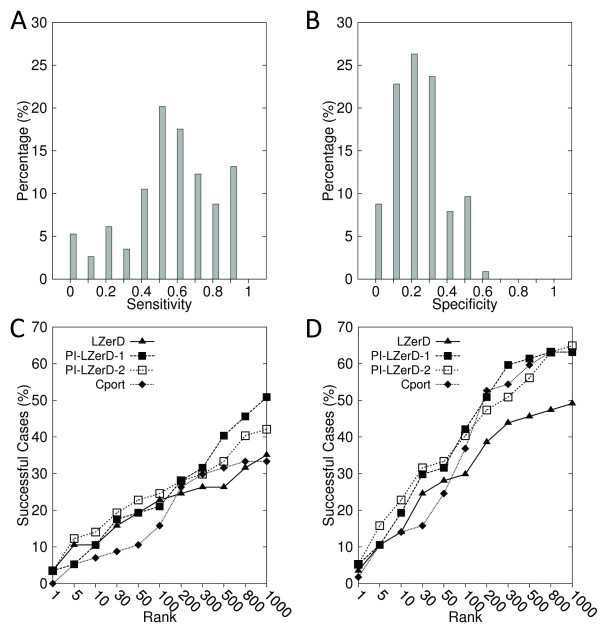
**Comparison of the docking prediction performance by PI-LZerD and CPORT**. The dataset contains 57 unbound proteins. The predictions were downloaded from the CPORT website. Distribution of **A**, the sensitivity and **B**, the specificity of the PPI site predictions by CPORT. **C**, hits within 2.5 Å iRMSD; **D**, hits within 4.0 Å iRMSD.

Finally, we compare PI-LZerD with a recently published related method, CPORT [74]. CPORT takes a consensus approach for PPI site prediction, combining six web servers. Predicted PPI site information is used for protein-protein docking in the framework the HADDOCK docking program. CPORT-HADDOCK translates predicted interface residues to what they call ambiguous interaction restraints (AIRs), which are distance restraints between provided (predicted) interface residues between a receptor and a ligand protein [14]. We used PPI site predictions of 57 unbound proteins that are made available as supplemental material of the paper at http://haddock.chem.uu.nl/services/CPORT. The distribution of the accuracy of the PPI site predictions is provided in Figures 10A &10B.

The performance of docking prediction with CPORT and PI-LZerD are compared in Figures 10C &10D. Overall, for both iRMSD threshold of 2.5 Å (Figure 10C) and 4.0 Å (Figure 10D), PI-LZerD-2 showed a higher success rate at each rank cutoff (x-axis). For example, PI-LZerD-2 obtained 14 success cases out of 57 complexes (24.6%) within 2.5Å when top 100 scoring decoys are considered, while CPORT had 9 successful cases (15.8%) at the same cutoff (Figure 9A). Using a 4.0 Å iRMSD threshold value, PI-LZerD-2 and CPORT obtained 23 (40.4%) and 21 successful cases (36.8%) within top 100 decoys, respectively.

## Conclusion

We have developed PI-LZerD, a pairwise docking algorithm that uses imperfect PPI prediction to improve docking accuracy. In the series of experiments, we showed that PI-LZerD successfully improved docking results even when accuracy of PPI information is significantly low. Unlike the post-filtering whose success largely depends on the accuracy of provided PPI information, PI-LZerD can use imperfect PPI prediction to improve prediction by exploring docking poses in the neighborhood of provided PPI prediction. PI-LZerD identifies matches of two proteins at local surface regions that only partially overlap with the provided PPI prediction. In addition, employing two iterations of docking searches (PI-LZerD-2) is shown to be more effective than one round of docking (PI-LZerD-1) because the two iterations enable exploring further from the provided PPI site prediction. Improvement of the average docking accuracy by PI-LZerD over LZerD was observed consistently in the series of benchmark experiments including docking using actual PPI site predictions as well as unbound docking cases.

While this work focused on pairwise docking, the same procedure can be applied for multiple protein-protein docking algorithms [94-100]. As the protein interactions and their networks have become a very important research focus in systems biology, the procedure developed here will be valuable for providing physical picture of such interactions.

## Authors' contributions

BL participated in design, implemented the algorithms, and drafted the paper. DK conceived of the study, participated in its design, and finalized the manuscript. All authors read and approved the final manuscript.

## Supplementary Material

Additional file 1 **Supplemental material for "Protein Docking Prediction Using Predicted Protein-Protein Interface"**. The file contains following five figures. Figure S1. Selecting decoys by the scoring function and/or by the cluster size. Figure S2. The procedure to compute "simulated" incorrect PPI site predictions. Figure S3. Docking prediction results using shifted PPI regions classified by the sensitivity of the PPI predictions. Figure S4. Docking prediction results using shifted PPI regions classified by the fnat of the PPI predictions. Figure S5. Comparison of prediction results using different numbers of decoys for running the second iteration of LZerD. Click here for file
