# Supplementary material for: Protein docking prediction using predicted protein-protein interface
Source: BMC Bioinformatics. 2012 Jan 10;13:7. doi: 10.1186/1471-2105-13-7 (PMC3287255; doi:10.1186/1471-2105-13-7)
Supplement: Additional file 1 — Supplemental material for "Protein Docking Prediction Using Predicted Protein-Protein Interface". The file contains following five figures. Figure S1. Selecting decoys by the scoring function and/or by the cluster size. Figure S2. The procedure to compute "simulated" incorrect PPI site predictions. Figure S3. Docking prediction results using shifted PPI regions classified by the sensitivity of the PPI predictions. Figure S4. Docking prediction results using shifted PPI regions classified by the fnat of the PPI predictions. Figure S5. Comparison of prediction results using different numbers of decoys for running the second iteration of LZerD. [file 1471-2105-13-7-S1.DOC]

Supplemental material for

**Protein Docking Prediction Using Predicted Protein-Protein Interface**

**BMC Bioinformatics**

Bin Li & Daisuke Kihara

Contact: [dkihara@purdue.edu](mailto:dkihara@purdue.edu)


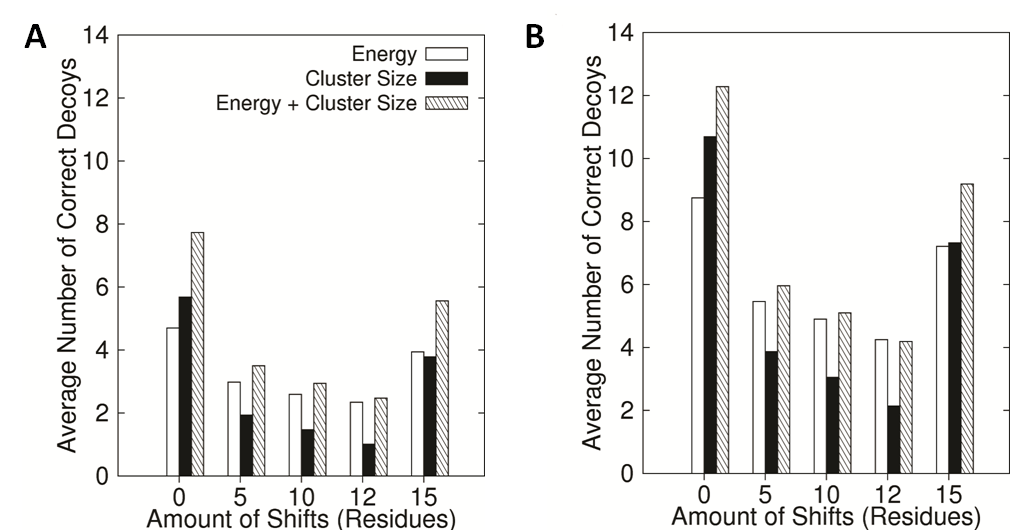


**Figure S1.** Selecting decoys by the scoring function and/or by the cluster size. The number of near native structures within the iRMSD of **A**, 2.5Å; **B**, 4.0Å; are shown among selected 60 decoys from the results of the modified LZerD run. The dataset of protein complexes with shifted PPI site information was used. Empty bars, the average number of hits among 60 decoys selected by considering the energy score value; filled bars, the selections was done by considering the cluster size; hatched bars, the combination of the energy score and the cluster size was considered for the selection. Modified LZerD was run with five different accuracy levels of PPI information, with correct (0 residue shift), 5, 10, 12, 15 residue shifted PPI information (x-axis).


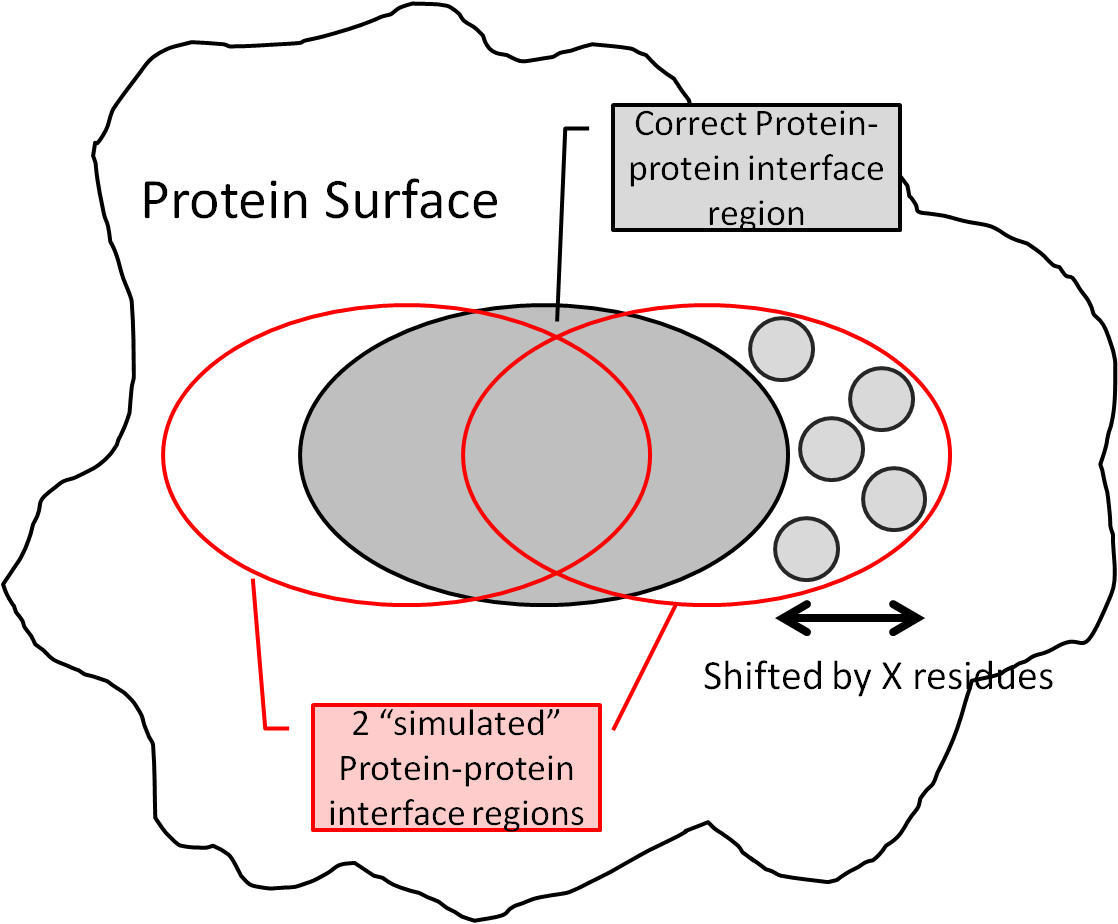


**Figure S2.** The procedure to compute “simulated” incorrect PPI site predictions. Given a correct PPI site (shown in gray), the longest axis of the PPI site is identified. Then, the correct PPI site is shifted by 5, 10, 12, or 15 residues to both directions along the axis. To make a shift, we removed X residues (5, 10, 12, or 15 residues) from one end of the correct PPI region and added the same number of residues to the opposite side of the PPI region. Therefore the shifting of PPI regions are done geometrically rather than along the protein sequence.


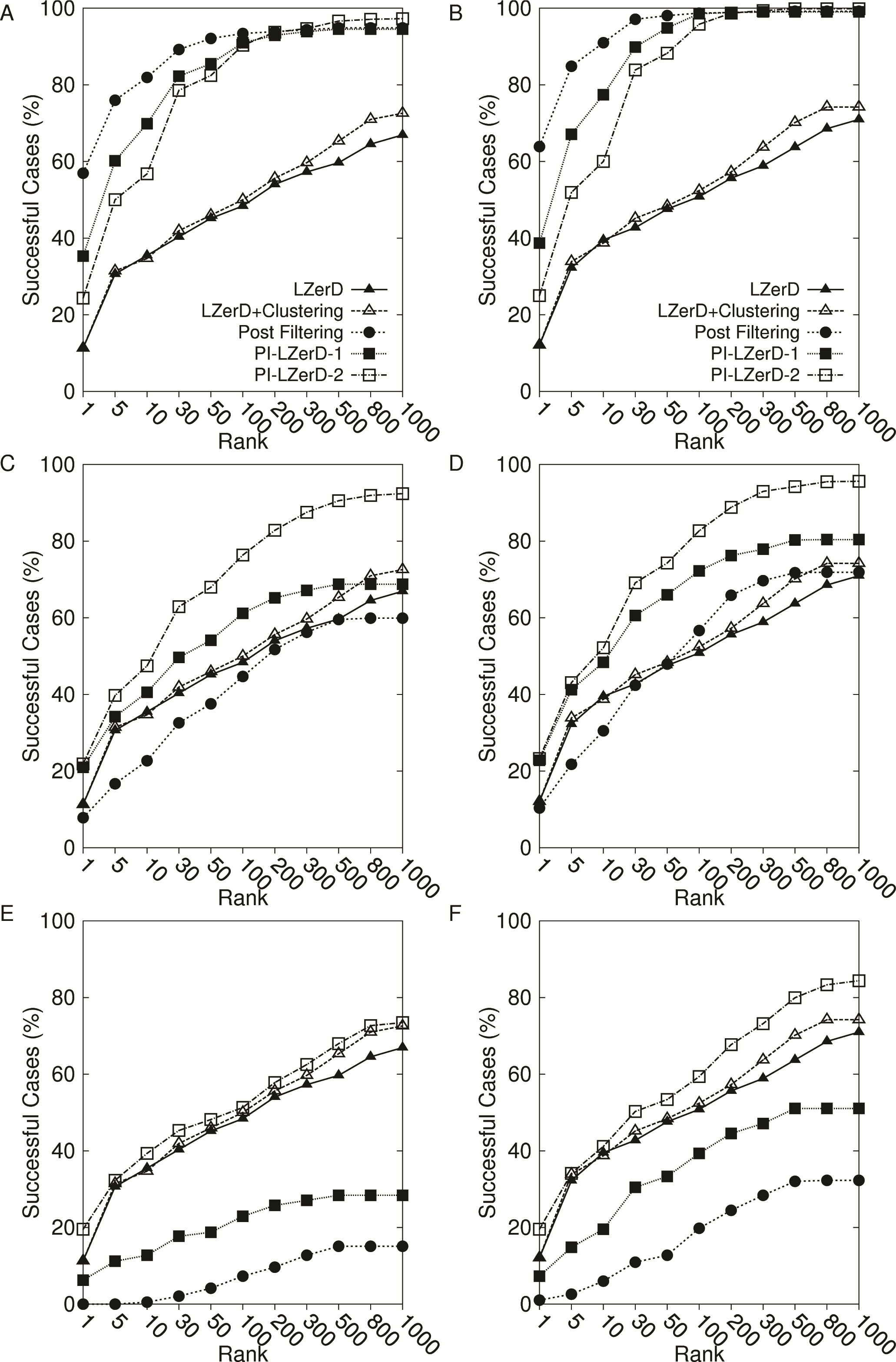


**Figure S3.** Docking prediction results using shifted PPI regions classified by the sensitivity of the PPI predictions. **A**, **B**, predictions using PPI regions of the sensitivity between 0.66 and 1.0; **C**, **D**, the sensitivity of the shifted PPI regions range from 0.33 to 0.66; **E**, **F**, the sensitivity range of the PPI regions is from 0.0 to 0.33. **A**, **C, E**, use an iRMSD of 2.5Å while **B, D, F,** use 4.0Å as the iRMSD threshold for correct predictions.


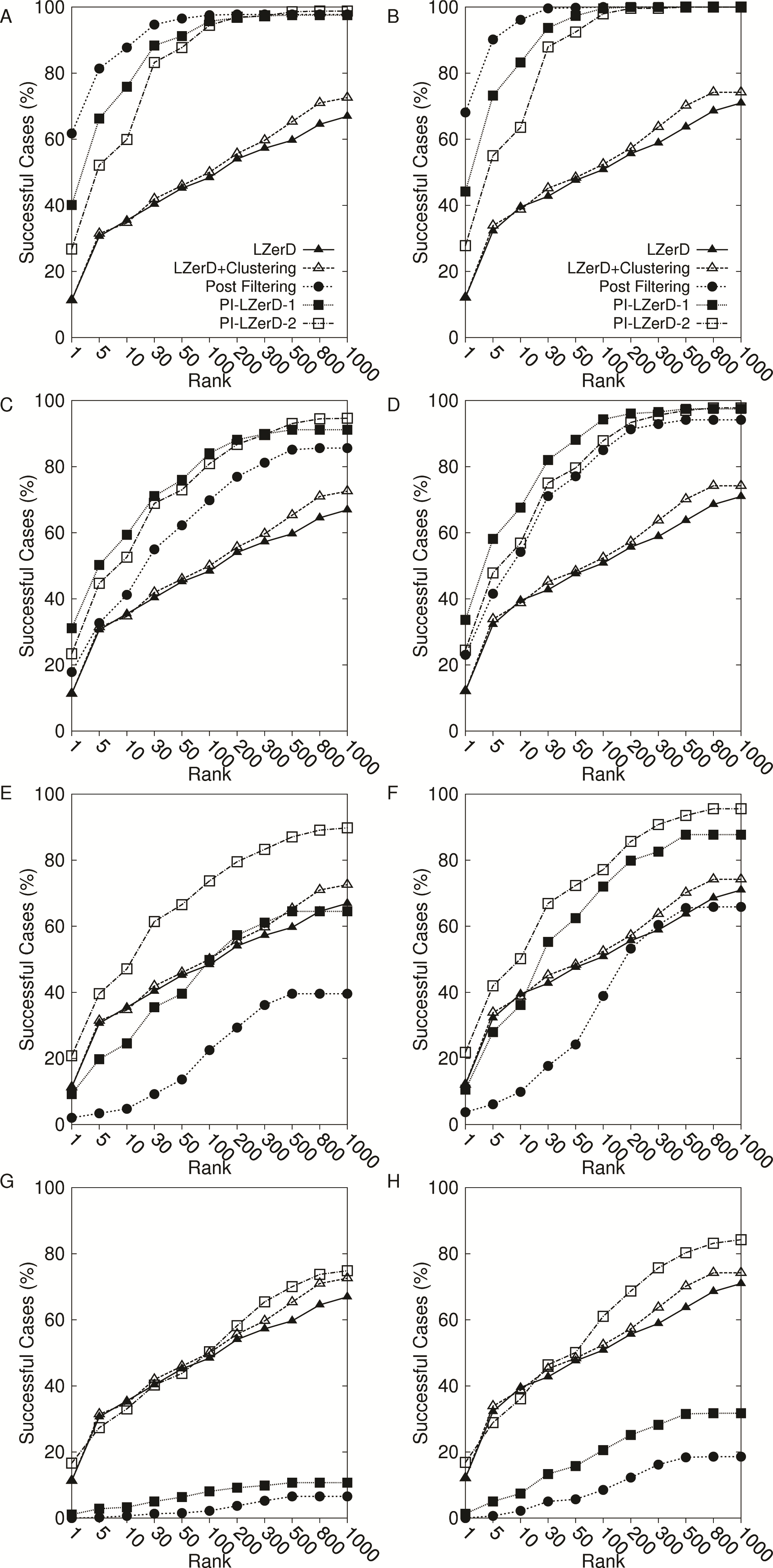


**Figure S4.** Docking prediction results using shifted PPI regions classified by the fnat of the PPI predictions. **A**, **B**, predictions using PPI regions of the fnat between 0.6 and 1.0 (489 complexes); **C**, **D**, fnat values range from 0.3 to 0.6 (633 complexes); **E**, **F**, the fnat range of the PPI regions is from 0.15 to 0.3 (293 complexes); **G, H**, fnat values range from 0.0 to 0.15 (457 complexes). In the parentheses, the number of complexes at each fnat range is shown. **A**, **C, E**, **G**, use an iRMSD of 2.5Å while **B, D, F, H,** use 4.0Å as the iRMSD threshold for correct predictions.


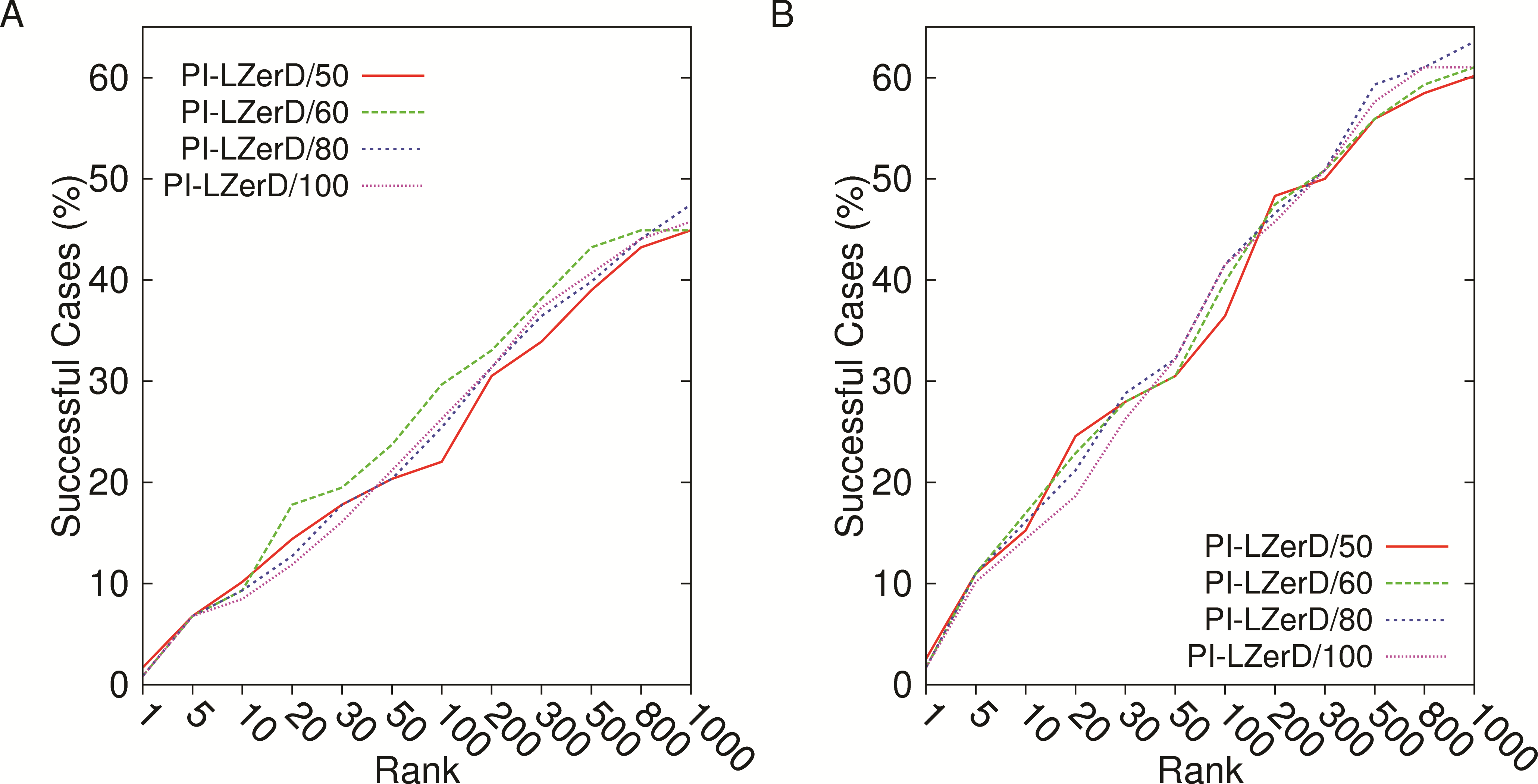


**Figure S5.** Comparison of prediction results using different numbers of decoys for running the second iteration of LZerD. The 118 unbound docking cases with predicted PPI sites by the meta-PPISP server were used. **A** and **B** show the % of the cases among 118 where a near native decoy within **A**, 2.5 Å; **B**, 4.0 Å; are obtained by PI-LZerD using 50 (red), 60 (green, originally reported), 80 (blue, dotted line), and 100 (purple, dotted line).
